# The kinetic energies of left ventricular 4D flow components correlate with established markers of prognosis and represent novel imaging biomarkers in both ischaemic and dilated cardiomyopathy

**DOI:** 10.1186/1532-429X-18-S1-O68

**Published:** 2016-01-27

**Authors:** Victoria Stoll, Aaron T Hess, Jonatan Eriksson, Petter Dyverfeldt, Tino Ebbers, Saul G Myerson, Carl Johan Carlhall, Stefan Neubauer

**Affiliations:** 1 grid.4991.50000000419368948OCMR, University of Oxford, Oxford, UK; 2 grid.5640.70000000121629922Division of Cardiovascular Medicine, Linköping University, Linköping, Sweden

## Background

Despite different aetiology of myocardial damage in dilated cardiomyopathy (DCM) and ischaemic cardiomyopathy (IHD), cardiac remodelling occurs in both, culminating in the end result of a dilated left ventricle with impaired function. Cardiac remodelling is a complex process in which numerous cellular, mechanical and flow processes become deranged. Insights into changes of multidimensional flow patterns and kinetic energy (KE) within the left ventricle are now afforded by the use of 4D flow CMR.

We hypothesised that greater derangements in 4D flow measures would relate to: 1) decreased mechanical cardiac function and dilatation, as assessed by LV ejection fraction (LVEF), LV myocardial strain and LV volumes, 2) increased levels of biochemical remodelling markers and 3) worsening patient symptoms and functional capacity. We hypothesised these changes to be independent of the initial aetiology of the myocardial damage, instead reflecting the self-propagating nature of cardiac remodelling.

## Methods

98 subjects (34 DCM patients, 30 IHD patients and 34 healthy controls) underwent CMR imaging for anatomy, tagging and 4D flow. The LV volume was analysed in 4 functional flow components; direct flow, delayed ejection flow, retained inflow and residual volume. The kinetic energy (KE) for these components was measured at end diastole.

## Results

**Figure 1:**
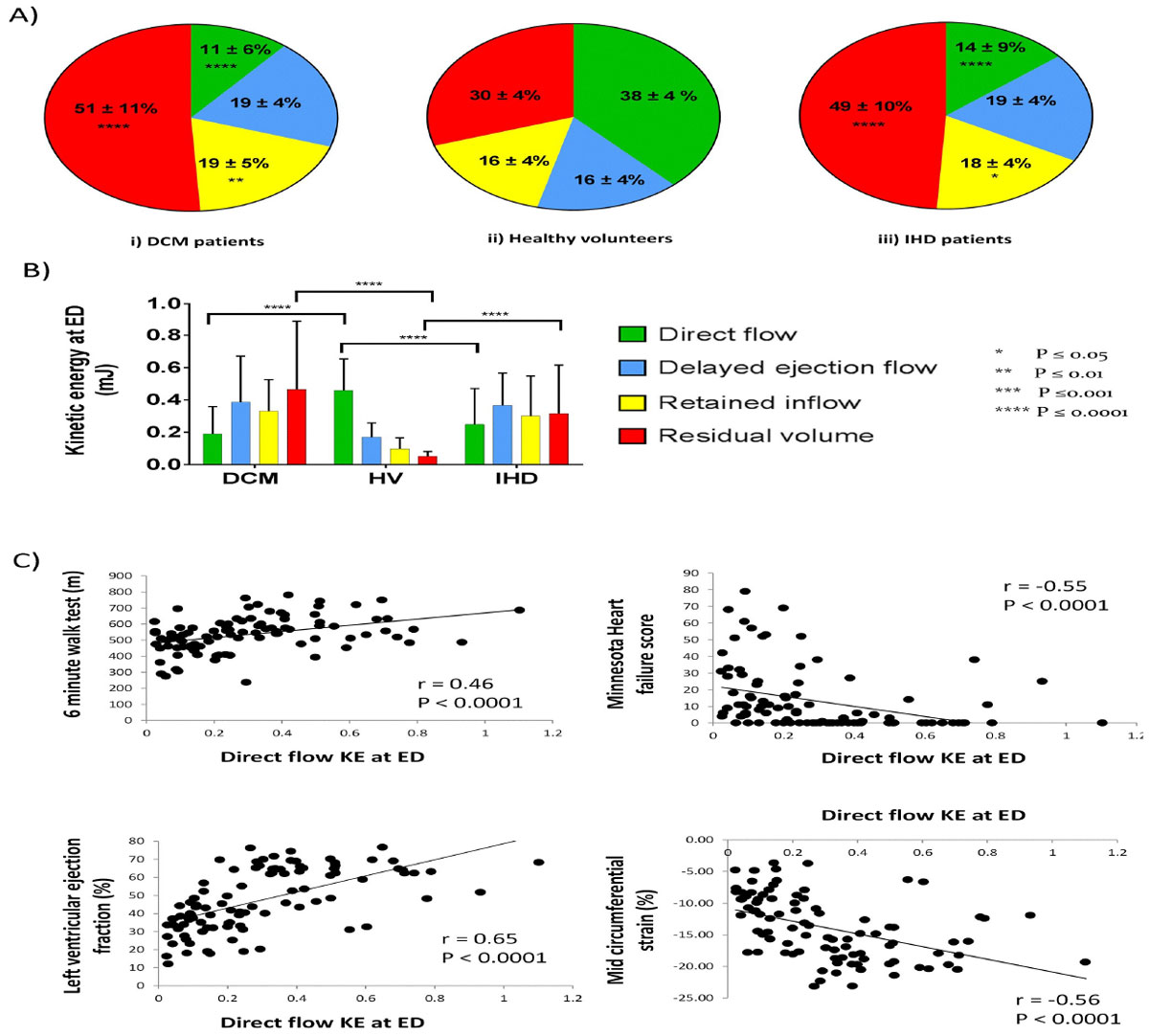
**A) The volume of the four flow components by mean percentage ± SD in relation to the end diastolic volume**. (P values calculated by ANOVA). B) Kinetic energy (KE) values for each flow component at end diastole. Bars show mean ± SD. The significant statistical differences for the KE values of the direct flow and residual volume components are highlighted between the patient groups and the healthy controls. (P values calculated by ANOVA for parametric data or Kruskal-Wallis for non-parametric data). C) Correlations between the direct flow component kinetic energy at end diastole and the 6 minute walk test, Minnesota heart failure score, LV ejection fraction and LV mid-circumferential systolic strain. (r calculated with Spearman's correlation).

Both patient groups had significantly decreased direct flow compared to controls (DCM 11 ± 6%, IHD 14 ± 9% vs 38 ± 4% P < 0.0001) and increased residual volume (DCM 51 ± 11%, IHD 49 ± 10% vs 30 ± 4% P < 0.0001) (fig 1A). The kinetic energy profile was significantly different for the direct flow (DCM 0.19 ± 0.17 mJ, IHD 0.25 ± 0.22 mJ vs 0.46 ± 0.19 mJ, P < 0.0001) and the residual volume (DCM 0.47 ± 0.43 mJ, IHD 0.32 ± 0.30 mJ vs 0.05 ± 0.03 mJ, P < 0.0001) between the patient groups compared to controls (fig 1B).

**Figure 2:**
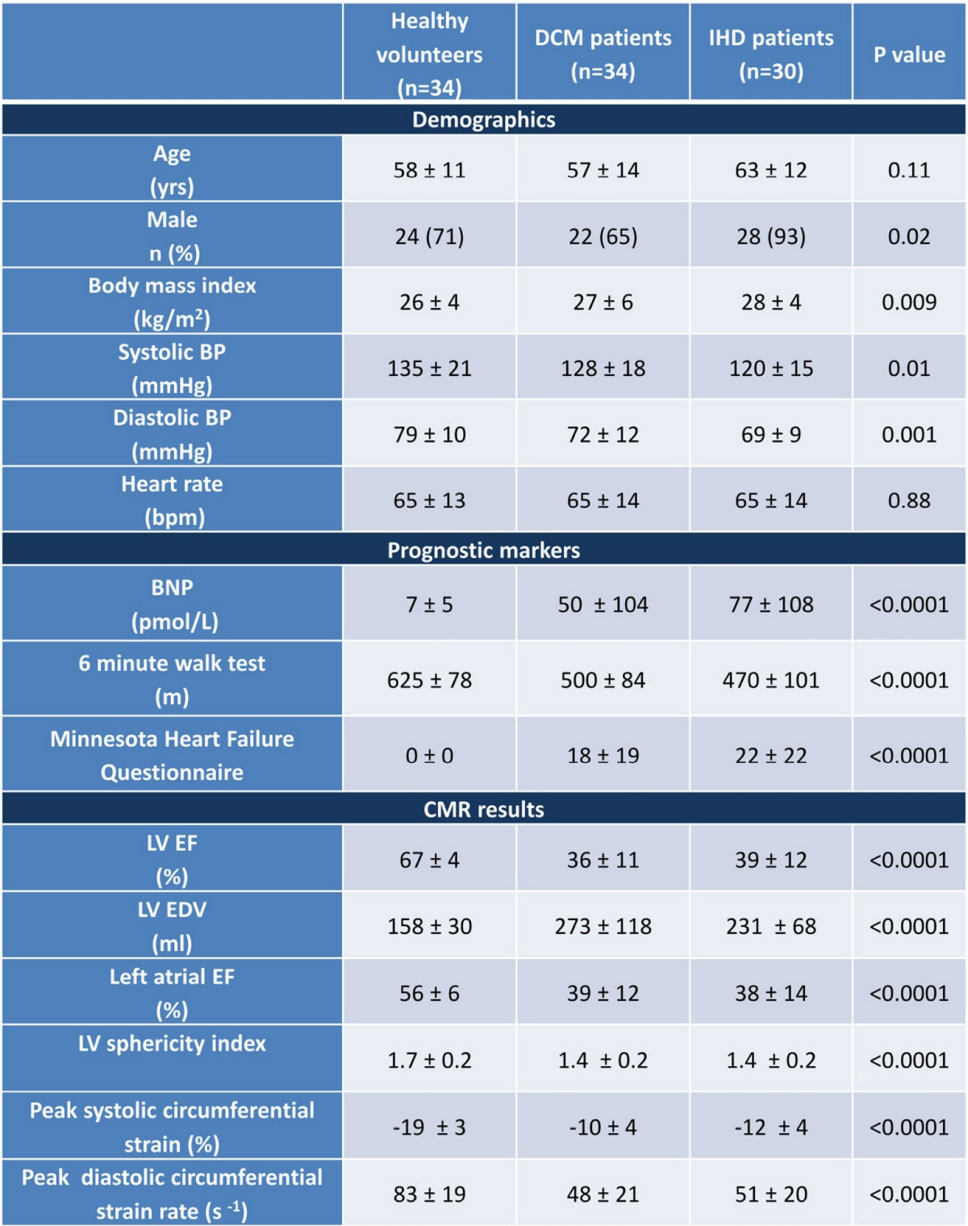
**Values are mean ± SD**. P values calculated by ANOVA for parametric data or Kruskal-Wallis for non-parametric data.

LVEF and strain were significantly impaired in patients vs controls (Figure 2). Direct flow KE correlated positively to the LVEF (r = 0.65), 6 minute walk test (6 MWT) (r = 0.46) and negatively to the LVEDV (r = -0.35), LVESV (r = -0.54), BNP (r = -0.45), LV strain (r = -0.56) and Minnesota HF questionnaire (MHFQ) (r = -0.55) (fig 1C). Residual volume KE correlated negatively to the LVEF (r = -0.87), 6 MWT (r = -0.49), and positively to the LVEDV (r = 0.79), LVESV (r = 0.89), BNP (r = 0.53),LV strain (r = 0.79) and MHFQ (r = 0.58). All P values for correlations P < 0.0001.

## Conclusions

Both DCM and IHD patients demonstrated less efficient blood flow patterns with deranged kinetic energy profiles compared to controls. The greater the derangement of flow parameters from normal, the worse the myocardial function, dilatation, BNP, 6 MWT and patient symptoms, irrespective of the initial pathology. This is the first study to suggest that derangements in 4D flow parameters are novel biomarkers of disease severity in cardiomyopathy patients and correlate with established markers of cardiac remodelling and prognosis, which may provide additive value in monitoring novel heart failure therapies and predicting prognosis.

